# The Woodrat Gut Microbiota as an Experimental System for Understanding Microbial Metabolism of Dietary Toxins

**DOI:** 10.3389/fmicb.2016.01165

**Published:** 2016-07-28

**Authors:** Kevin D. Kohl, M. Denise Dearing

**Affiliations:** ^1^Department of Biological Sciences, Vanderbilt University, NashvilleTN, USA; ^2^Department of Biology, University of Utah, Salt Lake CityUT, USA

**Keywords:** herbivory, host–microbe interactions, mammal, plant secondary compounds, plant–animal interactions

## Abstract

The microbial communities inhabiting the alimentary tracts of mammals, particularly those of herbivores, are estimated to be one of the densest microbial reservoirs on Earth. The significance of these gut microbes in influencing the physiology, ecology and evolution of their hosts is only beginning to be realized. To understand the microbiome of herbivores with a focus on nutritional ecology, while evaluating the roles of host evolution and environment in sculpting microbial diversity, we have developed an experimental system consisting of the microbial communities of several species of herbivorous woodrats (genus *Neotoma*) that naturally feed on a variety of dietary toxins. We designed this system to investigate the long-standing, but experimentally neglected hypothesis that ingestion of toxic diets by herbivores is facilitated by the gut microbiota. Like several other rodent species, the woodrat stomach has a sacculated, non-gastric foregut portion. We have documented a dense and diverse community of microbes in the woodrat foregut, with several genera potentially capable of degrading dietary toxins and/or playing a role in stimulating hepatic detoxification enzymes of the host. The biodiversity of these gut microbes appears to be a function of host evolution, ecological experience and diet, such that dietary toxins increase microbial diversity in hosts with experience with these toxins while novel toxins depress microbial diversity. These microbial communities are critical to the ingestion of a toxic diet as reducing the microbial community with antibiotics impairs the host’s ability to feed on dietary toxins. Furthermore, the detoxification capacity of gut microbes can be transferred from *Neotoma* both intra and interspecifically to naïve animals that lack ecological and evolutionary history with these toxins. In addition to advancing our knowledge of complex host-microbes interactions, this system holds promise for identifying microbes that could be useful in the treatment of diseases in humans and domestic animals.

## Introduction

Microbes represent the most abundant and diverse forms of life on earth (Hug et al., 2016). Environmental sampling techniques have revealed the vast diversity of microorganisms that were previously unknown through conventional culture techniques (Pace, 1997; Hug et al., 2016). Recently, we have begun to understand that animals are intricately associated with a diverse set of uncultivable microbes, mostly in their gastrointestinal tracts (Ley et al., 2008b). The cumulative genetic diversity encoded by the microbial symbionts (the “microbiome”) of humans is estimated to exceed that of the host by up to two orders of magnitude (Qin et al., 2010). Thus, hosts contain more genetic diversity, and likely more metabolic diversity, within their gut microbiota than within their own genome. This has given rise to the idea of the “holobiont”, i.e., that most organisms are actually collectives that function through their own genome in concert with the genomes of their associated microbes rather than as isolated individuals (Bordenstein and Theis, 2015; Theis et al., 2016). Indeed, microbes have been demonstrated to influence many aspects of animal performance, such as nutrition, immunity, and behavior (McFall-Ngai et al., 2013).

Gut microbes are thought to be especially important in the evolution of herbivory as a feeding strategy (Mackie, 2002). The major microbial service provided to hosts to facilitate herbivory is the fermentation of cellulose and other fibers that the animal hosts could not otherwise digest themselves. Through microbial fermentation, animal hosts obtain short-chain fatty acids (SCFAs) that can provide 30–70% of the daily energy requirements for some herbivores (Stevens and Hume, 2004). In addition to fermentation, gut microbes also provide other nutritional services to their hosts, such as the recycling of nitrogenous waste and the production of essential amino acids and vitamins (Stevens and Hume, 2004). The importance of these microbial partners to herbivore nutrition has resulted in the evolution of enlarged gut chambers in which to house them (Stevens and Hume, 2004). Additionally, as a group, mammalian herbivores have gut microbial communities that are distinct, more diverse, and enriched in metabolic pathways associated with synthesizing amino acids when compared to the gut microbial communities of omnivorous and carnivorous mammals (Ley et al., 2008a; Muegge et al., 2011).

Another potential service provided to herbivorous hosts by their gut microbiota is the degradation of ingested toxins. Plants defend themselves against herbivory through the production of myriad plant secondary compounds (PSCs), which can act as toxins, digestive inhibitors, and diuretics (Dearing et al., 2005). Herbivores have developed a number of physiological and behavioral strategies to overcome the challenges presented by PSCs, such as modified foraging patterns or enhanced liver detoxification (Dearing et al., 2005). It has long been proposed that herbivorous animals may also house gut microbes that aid in the metabolism of ingested toxins, thereby reducing the levels of toxins absorbed by hosts (Freeland and Janzen, 1974). Earlier studies on agricultural herbivores provided proof of concept that microbial detoxification could occur in natural plant-animal systems (Jones and Megarrity, 1986; Allison et al., 1992). More recently, this microbial function has been documented in several insect herbivores (Adams et al., 2013; Ceja-Navarro et al., 2015; Hammer and Bowers, 2015).

**FIGURE 1:**
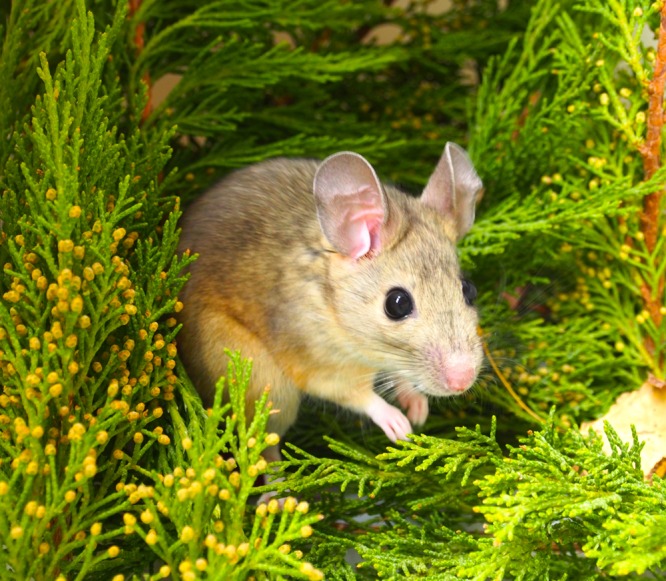
**A desert woodrat (*Neotoma lepida*) surrounded by terpene-rich juniper**.

To better understand the role of the microbiota in detoxification, while also evaluating the roles of host evolution and environment in sculpting such diversity, we have focused our work on numerous woodrat species within the genus *Neotoma* (**Figure 1**). This genus contains roughly 20 species of herbivorous rodents that are broadly distributed in the New World from the Arctic Circle to northern Central America (Edwards et al., 2001; Edwards and Bradley, 2002; Matocq, 2002; Patton et al., 2007). This genus is ideal as a model system because of the diversity of dietary strategies coupled with a well-documented evolutionary and dietary history. Numerous studies have documented the dietary specialization of woodrats (**Table 1**). Here, we review the body of work that we have conducted in this system of woodrats and their gut microbes, and highlight areas of future research.

**Table 1:** Details of dietary specialization in various woodrat species.

Woodrat Species	Location	Diet Breadth	Plant Species	Primary Class of Toxin	Reference for Woodrat Diet	Reference for Plant Chemistry
*N. stephensi*	Coconino County, Arizona	Specialist	One-seeded juniper (*Juniperus monosperma*)	Terpenes	Vaughan, 1982	Adams et al., 2014
*N. cinerea*	Atlin, British Columbia	Generalist	Arctic lupine (*Lupinus acticus*)	Alkaloids	Morton and Pereyra, 2008	Sharam and Turkington, 2005
*N. fuscipes*	Lassen County, California	Specialist	Incense cedar (*Calocedrus decurrens*)	Terpenes	McEachern et al., 2006	von Rudloff, 1981
*N. macrotis*^a^	Orange County, California	Specialist	Live oak (*Quercus agrifolia*)	Phenolics	Atsatt and Ingram, 1983	Atsatt and Ingram, 1983
*N. bryanti*^b^	Orange County, California	Generalist	Cactus (*Opuntia* spp.)	Oxalate	Atsatt and Ingram, 1983	Justice, 1985
*N. lepida*	Washington County, Utah	Specialist	Creosote (*Larrea tridentata*)	Phenolics	Karasov, 1989	Mabry et al., 1977
*N. lepida*	Tooele County, Utah	Specialist	Utah juniper (*Juniperus osteosperma*)	Terpenes	Skopec et al., 2015	Adams et al., 2014
*N. lepida*	Death Valley, California	Specialist	Honey mesquite (*Prosopis glandulosa*)	Alkaloids	Smith et al., 2014	Samoylenko et al., 2008
*N. devia*	Coconino County, Arizona	Specialist	Mormon tea (*Ephedra* spp.)	Alkaloids	Dial, 1988	Nielsen et al., 1927
*N. albigula*	Grand County, Utah	Specialist	Cactus (*Opuntia* spp.)	Oxalate	Kohl et al., 2014a	Justice, 1985

*^a^*N. macrotis* was previously identified as *N. fuscipes*. Species reclassified by Matocq (2002). ^b^*N. bryanti* was previously identified as *N. lepida*. Species reclassified by Patton et al. (2007).*

## Woodrats as a Tractable System for Gut Microbial Ecology

To empirically study many aspects of microbially aided detoxification, woodrats must be housed in captivity to feed them experimental diets and monitor various physiological parameters. Numerous studies have documented differences between the gut microbial communities of wild animals and their captive counterparts (Uenishi et al., 2007; Scupham et al., 2008; Villers et al., 2008; Xenoulis et al., 2010; Wienemann et al., 2011). Thus, we were concerned whether woodrats would lose their ecologically relevant gut microbiota when brought into captivity. To address this concern, we collected fecal samples from woodrats in the wild and over a time series in captivity. We confirmed that feces collected from traps in nature were representative of aseptically collected samples (Kohl et al., 2015). Woodrats retained a majority (>60%) of their natural gut microbes, even after 6 months in captivity (Kohl and Dearing, 2014). Potential environmental sources of microbes in captivity (such as rabbit chow, bedding, etc.) contributed minimally to the microbiota of captive woodrats (Kohl and Dearing, 2014). Quite remarkably, woodrats retained their individual ‘microbial signatures’ when brought into captivity, suggesting that a captive lifestyle does not homogenize microbial diversity across individuals or species (Kohl and Dearing, 2014; Kohl et al., 2014c). Thus, we conclude that studies involving woodrats in captivity are still ecologically relevant.

It should be noted that the microbial communities of wild and captive woodrats were not identical. Captive woodrats harbored distinct gut microbiota when compared to animals in nature, with lower diversity and differential abundances of some microbial taxa (Kohl and Dearing, 2014; Kohl et al., 2014c). It is unclear what underlies this loss of diversity. The most obvious explanation would be a change in their diet, given that diet can rapidly alter gut microbial communities (David et al., 2014). However, when the dietary specialist, *Neotoma stephensi*, was returned to a diet of 75% juniper (its native diet) after 6 months in captivity, none of the lost microbial diversity was rescued (Kohl et al., 2014c). Thus, in this experiment there appeared to be permanent loss of some microbial members. Provision of the woodrats’ natural diets upon entrance to captivity may be critical in maintaining the natural gut flora compared with a reintroduction of the diet at a later time. It would be interesting to compare the effects of captivity *per se* on the gut microbial communities of woodrats by examining the microbiota of woodrats fed their natural diets upon entrance into captivity compared to those immediately fed laboratory diets.

Woodrats are also especially interesting from a microbial perspective because of their distinct gut anatomy. Most rodents are hindgut fermenters. In accordance with this notion, woodrats have large, fermentative cecal chambers in their hindguts that compose roughly 6% of their body mass (Skopec et al., 2008; Kohl et al., 2014b). However, in addition to this hindgut chamber, woodrats exhibit semi-segmented stomach morphology and harbor a foregut chamber proximal to their gastric stomach (Carleton, 1973; Kohl et al., 2014b). Although this foregut chamber only composes ∼2% of their body mass, it contains remarkable microbial density and diversity. The microbial density of the foregut chamber is on par with that of the cecum (10^10^ live microbial cells/g contents), a segment of the gut is known to play an important role in housing microbes, particularly bacteria. In addition, the foregut exhibits higher concentrations of microbial products (short chain fatty acids and ammonia nitrogen) than the cecum (Kohl et al., 2014b). Thus, woodrats maintain a dense and active microbiota in the foregut.

The function of the rodent foregut chamber has puzzled mammalogists for over a century (Toepfer, 1891; Carleton, 1973). The residence time of food material in this chamber is less than 1.5 h, which is not long enough for extensive fiber fermentation (Kohl et al., 2014b). We propose that this chamber may have another role: that of microbial detoxification. Detoxification in this chamber would allow for the metabolism and subsequent inactivation of PSCs early on in the digestive tract, before absorption in the small intestine. This idea is in agreement with the hypothesis that the rumen evolved first for microbial detoxification and was later used for cellulolytic fermentation (Hume and Warner, 1980; Mackie, 2002).

## Evidence for Microbial Detoxification in Woodrats

We have taken several approaches to investigate whether microbes in the gut have the capacity to metabolize ingested plant toxins. The first piece of evidence along these lines stemmed from the detection and identification of microbes capable of this function. We employed sequencing-based approaches (of the 16S rRNA gene) to inventory the gut microbial communities of several woodrat species. These studies have demonstrated the presence of numerous gut microbes implicated in detoxification of various compounds (**Table 2**). Additionally, for a limited set of woodrat species and classes of PSCs, we have used culture-based techniques to isolate microbes capable of degrading tannins (Kohl et al., 2016) and oxalate (Miller et al., 2014) (**Table 2**) and have measured their capacity for these functions (Miller et al., 2014; Kohl et al., 2016).

**Table 2:** Summary of evidence for detoxifying microbes in the woodrat gut.

Microbial taxa	Woodrat species	Method of detection	Class of PSC that taxa is capable of degrading	Putative or demonstrated PSC metabolism in woodrats?	Citation for detection	Citation for microbial degradation ability (for putative only)
*Coprococcus*	*N. bryanti, N. albigula*	16S rRNA sequencing	Phenolics	Putative	Kohl et al., 2011; Miller et al., 2016	Patel et al., 1981
*Lactobacillus*	*N. lepida, N. bryanti, N. albigula*	16S rRNA sequencing	Phenolics	Putative	Kohl et al., 2011; Kohl and Dearing, 2012; Miller et al., 2016	Shimada et al., 2006
*Oxalobacter*	*N. albigula*	16S rRNA sequencing	Oxalate	Putative	Miller et al., 2014	Allison et al., 1985
*Bacillus*	*N. lepida*	Culture techniques	Phenolics (tannins)	Demonstrated	Kohl et al., 2016	
*Enterococcus*	*N. lepida*	Culture techniques	Phenolics (tannins)	Demonstrated	Kohl et al., 2016	
*Escherichia*	*N. lepida*	Culture techniques	Phenolics (tannins)	Demonstrated	Kohl et al., 2016	
*Clostridium*	*N. albigula*	Culture techniques	Oxalate	Demonstrated	Miller et al., 2014	
*Enterococcus*	*N. albigula*	Culture techniques	Oxalate	Demonstrated	Miller et al., 2014	
*Lactobacillus*	*N. albigula*	Culture techniques	Oxalate	Demonstrated	Miller et al., 2014	

We have also demonstrated that consuming PSCs sculpts the community structure of the woodrat gut microbiota. For example, certain populations of *N. albigula* specialize on cactus, thereby ingesting a diet high in oxalate (**Table 1**). Increasing the concentration of oxalate in diets fed to captive *N. albigula* altered the composition of the gut microbiota (Miller et al., 2016). Specifically, animals fed higher concentrations of oxalate harbored higher concentrations of known oxalate-degrading bacteria, such as *Oxalobacter* spp. and several other taxa (Miller et al., 2016). As oxalate is only degraded by microbial metabolism, these studies provide strong evidence that the microbiota is responding to an ingested dietary toxin.

We found a similar outcome in an independent system consisting of two species of woodrats, *N. lepida* and *N. bryanti*, in populations that feed on creosote leaves. Creosote (*Larrea tridentata*) produces a phenolic-rich resin on its leaves that is both chemically complex and abundant (Mabry et al., 1977). When captive *N. lepida* and *N. bryanti* were fed a diet amended with creosote resin, the microbiota in the woodrat foregut was significantly altered compared to the communities in animals ingesting a diet lacking creosote resin (Kohl and Dearing, 2012). Interestingly, the responses of the gut microbial community are dependent on previous ecological and evolutionary experience with creosote bush. The abundance of Actinobacteria increased in response to creosote resin in the gut communities of individuals of *N. lepida* and *N. bryanti* that typically occur with and feed on creosote (Kohl and Dearing, 2012). Actinobacteria is a phylum well known for its biotransformation abilities and thought to be important in the degradation of plant phenolics in the termite gut (Donova, 2007; Le Roes-Hill et al., 2011). However, creosote resin did not elicit an increase in Actinobacteria abundance in populations of *N. lepida* and *N. bryanti* that do not overlap with creosote bush, and consume other plant species (Kohl and Dearing, 2012). Thus, we hypothesize that the microbial communities of herbivores are specifically adapted to the PSCs that herbivores consume.

Our work has also uncovered an interesting interaction between PSC consumption and microbial diversity. For example, the microbial communities of *N. albigula* increase in diversity with increasing levels of dietary oxalate (Miller et al., 2016). Similarly, the addition of phenolic-rich creosote resin in diets fed to *N. lepida* and *N. bryanti* increases metrics of microbial diversity in woodrats from populations that have previous ecological and evolutionary experience with this PSC (Kohl and Dearing, 2012). However, woodrats from populations of *N. lepida* and *N. bryanti* that lack this ecological and evolutionary experience with creosote bush exhibit decreases in gut microbial diversity when they are fed diets containing creosote resin compared to diets lacking resin (Kohl and Dearing, 2012). In general, taxonomic diversity of a microbial community is correlated with functional diversity (Human Microbiome Project Consortium, 2012), and so PSCs might increase microbial functions in adapted microbial communities, but not in naïve communities. The interactions between PSCs, evolutionary experience, and microbial diversity remain to be further studied.

The notion that gut microbes facilitate the ingestion of high doses of plant compounds by herbivores cannot be unequivocally demonstrated simply by the isolation of PSC-degrading microbes or alterations in community structure. Furthermore, it could be argued that the capacity of these microbes to metabolize PSCs is not great enough to reduce the impacts of toxins on the herbivore host by physiologically meaningful levels. To address these issues, we have performed several performance-based studies to demonstrate the importance of gut microbes in allowing herbivores to consume PSCs. These studies have largely been focused on *N. lepida*, which as explained earlier, has populations of woodrats with ecological and evolutionary experience with creosote bush and its phenolic-rich resin (“experienced” populations), as well as other populations that lack this experience with creosote (“naïve” populations). Importantly, when animals from these two populations are brought into captivity, they exhibit differential tolerance to creosote PSCs, such that the “experienced” population can consume 25% more creosote resin (Mangione et al., 2000). We first demonstrated a role of the microbiota in creosote tolerance by disrupting the microbiota of “experienced” woodrats with oral antibiotics, which significantly impaired the woodrats ability to consume diets with creosote resin (Kohl et al., 2014d). We also conducted microbial transplants from “experienced” woodrats into “naïve” woodrats, and significantly increased the ability of “naïve” woodrats to consume creosote resin (Kohl et al., 2014d). Microbial inventories of the “naïve” recipients confirmed that the transplants were effective at introducing a community structure of the gut microbiota that was more similar to that found in “experienced” woodrats (Kohl et al., 2014d). An additional study demonstrated that the transplantation of the “experienced” woodrat microbiota into lab rats significantly increased their ability to consume tannic acid, a phenolic PSC (Kohl et al., 2016). Thus, gut microbes play a critical and essential role in facilitating the ingestion of PSCs by mammalian herbivores.

## Mechanisms of “Microbial Facilitation” for Consuming PSCs

There are several potential mechanisms through which gut microbes facilitate the ingestion of dietary toxins. The most likely is direct metabolism of these compounds in the gut, prior to absorption into the blood stream. This is clearly the case for the degradation of oxalate in the gut of *N. albigula*, as mammals do not produce enzymes capable of degrading this compound (Justice, 1985; Miller et al., 2016). Several isolated bacteria from the woodrat gut contain the oxalyl-CoA decarboxylase gene, a key enzyme in oxalate catabolism (Miller et al., 2014). There is also evidence for the concept of direct degradation of phenolics by gut microbes in woodrats. When *N. lepida* with experience to creosote are fed resin, the abundances of genes associated with the metabolism of aromatic compounds increases in the metagenome, particularly the gene encoding for aryl-alcohol dehydrogenase (Kohl et al., 2014d).

**FIGURE 2:**
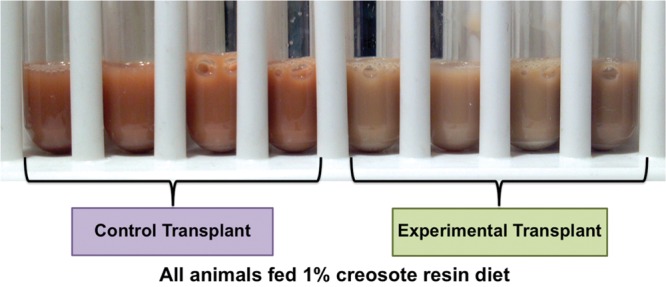
**Urine collected during the microbial transplant experiment described in Kohl et al. (2014d)**.

In addition to the identification of particular genes for detoxification, further evidence for microbial degradation can be observed with metabolomic approaches. If microbes were directly detoxifying PSCs in the gut, lower concentrations of PSCs should be absorbed into the blood stream, where they are acted on by the liver and excreted in the urine (Forbey et al., 2013). Therefore, we compared urine composition between “naïve” woodrats given the microbiota from “experienced” woodrats and “naïve” woodrats, all of which were being fed diets containing phenolic-rich creosote resin. Although these two groups were consuming the same doses of creosote resin (Kohl et al., 2014d), the urine was visually distinctive between the two groups (**Figure 2**). The urine of the “naïve” woodrats with their native microflora had a red color, commonly produced when these woodrats consume high doses of creosote resin (**Figure 2**). However, the “naïve” woodrats that received a transplant of “experienced” microbiota produced brown urine, characteristic of what “experienced” woodrats produce. Additionally, the urine of woodrats that received an “experienced” microbiota was less acidic, indicative of less reliance on host detoxification (Foley et al., 1995; Kohl et al., 2014d). Last, metabolomic analysis detected urinary metabolites of creosote resin that were uniquely produced in the animals that received the microbial transplant, suggesting that the “experienced” microbiota changes the detoxification routes of PSCs (Kohl et al., 2014d). The use of isotopically- or radio-labeled tracer molecules may help to better understand the capacity for direct detoxification in the gut.

Further, gut microbes may impact how hosts themselves absorb or metabolize PSCs. For example, several gut microbes induce expression of multidrug resistance protein-1a (*mdr1a*) in intestinal tissue (Hooper et al., 2001). These proteins are expressed along the gut lining, and transport absorbed compounds back into the gut lumen, thus limiting systemic absorption (Dearing et al., 2005). Differential microbial communities across woodrat populations might impact host gene expression of this enzyme, and thus absorption rates of PSCs. Additionally, germ-free mice (those lacking gut microbial population) exhibit lower expression of detoxification enzymes in intestinal and liver tissues when compared to mice with gut microbial communities (Hooper et al., 2001; Claus et al., 2011). Even in mice with a gut microbial community, differential community compositions may influence hepatic detoxification. For example, relative abundances of several members of Coriobacteriaceae in the gut correlate with Cyp3a11 enzyme activities in liver tissue (Claus et al., 2011). When diet was controlled for, the “naïve” and “experienced” woodrats exhibit differential hepatic gene expression profiles (Malenke et al., 2014). These differences might be driven by disparate microbial communities found in these two populations coupled with interactions with hepatic gene expression. However, the current evidence for this mechanism in woodrat gut communities is preliminary and circumstantial.

Another mechanism by which gut microbes might facilitate ingestion of toxins is by maintaining nutritional functions even when hosts consume diets rich in PSCs. In naïve animals, such as sheep and rats, the addition of PSCs to diets significantly decreases the digestibility of fiber and nitrogen (Adams et al., 1992; Dawson et al., 1999). This decrease in microbial function is likely due to the antimicrobial and inhibitory properties of many PSCs (Encarnación and Garcia, 1991). However, “experienced” woodrats are able to maintain high digestive performance even on diets containing PSCs (Meyer and Karasov, 1989; Skopec et al., 2008). It could be that the gut microbes of “experienced” woodrats are adapted to the PSCs in their native diets, and so are able to continue providing nutritional benefits to woodrats even in the face of high doses of toxins. However, in the microbial transplant experiment, woodrats that received the “experienced” microbiota defended body mass better, despite similar food intake and dry matter digestibility. These results suggest nutritional roles do not solely explain the differences in toxin tolerance. Rather, other physiological costs, such as detoxification, which is energetically expensive for woodrats, were likely responsible for the differential body mass (Sorensen et al., 2005; Kohl et al., 2014d). Overall, how PSCs might impact the nutritional services of the woodrat gut microbiota remain to be further explored.

## Implications

Our body of work represents, to our knowledge, the first to demonstrate that gut microbes facilitate the ingestion of PSCs in wild mammalian herbivores. While our work is conducted in rodents, we believe our findings have implications and relevance for understanding the role that gut microbes play in facilitating herbivory in other mammal species. Herbivory is the most common feeding strategy among mammals (Price et al., 2012), and herbivorous mammals can have large impacts on overall ecosystem structure (Martin and Maron, 2012). Thus, natural scenarios might exist where gut microbes dictate the foraging strategies of a mammalian herbivore, which then scale up to impact the structure of an entire ecosystem. Additionally, under current patterns of global climate change and changing patterns of human land-use practices, wild herbivores may be faced with higher concentrations, different classes, or more potent PSCs (Coley, 1998; Verhoeven et al., 2009; Kurnath et al., 2016). Acquiring novel gut microbes to aid in detoxification may represent a rapid route of ecological adaptation to cope with these new toxic challenges.

Gut microbes with a detoxification function in woodrats may also serve the agricultural industry. There is interest in developing probiotics of tannin-degrading bacteria that could be inoculated into agricultural herbivores to increase growth and feed conversion efficiency (Kumar et al., 2014). We have demonstrated that tannin-degrading bacteria isolated from woodrats can be transplanted to lab rats and increase their tolerance to dietary tannins (Kohl et al., 2016). Additionally, there is interest in generating methods that allow small ruminants to consume terpene-rich junipers (Utsumi et al., 2013), alkaloid-rich grasses (Catanese et al., 2014), and phenolic-rich oaks (Silanikove et al., 1996). Given the large diversity of PSC classes that woodrats consume (**Table 1**), these rodents may be rich sources of novel toxin-degrading microbes that could be used in the agricultural industry.

The work on the microflora of woodrats and other herbivores also has relevance for human health. The gut microbes of woodrats are capable of degrading compounds that are detrimental to human health. For example, oxalate is common in human diets and cannot be metabolized by human enzymes. It is the leading component of kidney stones, and therefore, in humans with kidney stone disease, it is considered a harmful dietary compound that should be consumed sparingly (Miller and Dearing, 2013). However, oxalate can be metabolized by gut microbes. The diverse gut microbiota of the white-throated woodrat has a tremendous capacity for degrading oxalate (Miller et al., 2014). Our preliminary work suggests that the function of this microbial community can be transplanted from woodrats to other species, and thus this community holds potential for development as a highly effective probiotic in humans. Since oxalate is common in plants, the microbial communities of other mammalian herbivores may offer additional possibilities for probiotic therapy.

In addition to degrading detrimental dietary compounds, gut microbes are also capable of degrading beneficial compounds, such as pharmaceuticals. This interaction between the gut microbiota and drugs may alter their efficacy of prescribed compounds (Haiser et al., 2014). Evolutionarily, these microbial pathways were likely first targeted toward plant compounds, given that many prescribed drugs are actually plant-derived (Saklani and Kutty, 2008). Thus, understanding the mechanisms by which the gut communities of woodrats and other wild herbivores metabolize PSCs may facilitate the development of microbial screening assays as part of a personalized medicine approach to better predict drug efficacy in individual patients.

In summary, we are only beginning to unravel the complex interactions that occur between herbivores, their gut microbial symbionts, and ingested toxins. Understanding these interactions will advance our knowledge of ecological interactions in general. In addition, this knowledge may be applicable to issues related to society, including human health and agriculture practices.

## Author Contributions

KDK and MDD co-wrote the paper and approved it for publication.

## Conflict of Interest Statement

The authors declare that the research was conducted in the absence of any commercial or financial relationships that could be construed as a potential conflict of interest.
